# Dr. Wm. H. Dwinelle—Fifty Years

**Published:** 1889-09

**Authors:** 


### Dr. Wm. H. Dwinelle—Fifty Years.

At the recent meeting of the American Dental Association it was the good fortune of those who were present to take by the hand and greet Dr. William H. Dwinelle, of New York City, and it was a great pleasure to see him so well and strong, both physically and mentally. Time has dealt kindly with him; he has a vigor that but few men can hope to attain. And this notwithstanding he has been in the active practice of dentistry for over fifty years, having begun practice, after a sufficient pupilage, in February, 1839. He has during all this time been a hard worker, in the full practice of his profession. Perhaps no man in the practice of dentistry in this country has applied himself so closely, and done as much hard work as Dr. Dwinelle; and not only in the practical part has he stood foremost, but in that which pertains to the science, he has ever been an earnest and industrious worker; and especially was this true in the early history of the profession when such work was most needed. And of the character of the work, it need only be said, that what he then wrote and said, stands to-day as true and uncontroverted as at the time it was uttered. It is remarkable that the progress of the times has not made it necessary for him to revise or much change what he then wrote or spoke. His professional standard was always a very high one, both in a practical and scientific aspect.

He was one amongst the first to demonstrate the use and value of the microscope in investigations in respect to dentistry. He took a great interest in, and did much for the improvement and devising of appliances and instruments for the dentist's use, and that, too, at a day when such things were most needed and most important. The manufacturers of forty years ago, could they now speak, would have much to say on this subject; much more than we can now say.

He has ever been, from the beginning of his career to the present time, one of the best operators in this country; he has very few equals and no superiors as a uniformly good operator. Dr. Dwinelle's judgment and discrimination in respect to professional matters has always been remarkable, and this has been especially shown in his ability to promptly recognize and decide upon the merits of any new instrument, appliance, or material suggested or presented for use or favor. The presence and the impression he made upon the membership of the association was the occasion of the passage, by a unanimous vote, of a resolution to the effect that any one having been for fifty years in the active and honorable practice of dentistry, shall be made a life member with the remittance of annual dues.

And now following this we will venture the suggestion that any one who has pursued the active practice of dentistry in an honorable manner, and in such a way as to promote the interest of his profession, is entitled to a public recognition in some suitabe manner by his professional brethren. What the form of such a recognition should be we will not now attempt to define. But if the matter should meet with favor it will soon assume a feasible form. Now it is in order for somebody to second the motion. We hope ere long to publish a biographical sketch of Dr. Dwinelle.

